# Associations between adoption of eHealth management module and optimal control of HbA1c in diabetes patients

**DOI:** 10.1038/s41746-023-00807-w

**Published:** 2023-04-13

**Authors:** Junjie Huang, Sze Chai Chan, Samantha Ko, Ellen Tong, Clement S. K. Cheung, Wing Nam Wong, Ngai Tseung Cheung, Martin C. S. Wong

**Affiliations:** 1 JC School of Public Health and Primary Care, Faculty of Medicine, Chinese University of Hong Kong, Hong Kong SAR, China; 2 Centre for Health Education and Health Promotion, Faculty of Medicine, Chinese University of Hong Kong, Hong Kong SAR, China; 3 Information Technology and Health Informatics Division, Hospital Authority, Hong Kong SAR, China; 4 School of Public Health, The Chinese Academy of Medical Sciences and Peking Union Medical College, Beijing, China; 5 School of Public Health, Peking University, Beijing, China

**Keywords:** Software, Public health

## Abstract

In January 2021, the eHealth App was launched in Hong Kong by the Hong Kong government to support the Electronic Health Record Sharing System (eHRSS). A Health Management Module in the eHealth App introduced new functions to record blood pressure, blood sugar, and heart rate, and downloading and sharing records. This study aims to compare the level of glycaemic control between users of the eHealth App and non-users. Type 2 diabetes patients who have joined the eHRSS with existing haemoglobin A1c (HbA1c) level records are recruited. Correlations between predictors and optimal HbA1c control (<7%) are examined using logistic regression analyses. A total of 109,823 participants are included, with 76,356 non-users of eHealth App, 31,723 users of eHealth App, and 1744 users of the eHealth Management Module together with the App. We collect HbA1c values from Jan 2021 to May 2022, and they are 6 months after the use of the App on average. Users of the eHealth Management Module are found to have more optimal HbA1c levels across all subgroups, with the strongest effect observed in younger females (aOR = 1.66, 95% CI = 1.27–2.17). eHealth App usage is also positively associated with optimal HbA1c levels, particularly amongst younger females (aOR = 1.17, 95% CI = 1.08–1.26). Overall, users of eHealth App and eHealth Management Module demonstrate more optimal HbA1c levels when compared with non-users, particularly among younger adults and females. These findings support its potential adoption in diabetes patients. Future studies should examine the impact of eHealth interventions on other clinical targets and diabetes complications.

## Introduction

The ongoing efforts to integrate technological platforms to improve the delivery of healthcare services has culminated in the development of electronic-health (eHealth) applications and tools. eHealth aims to make use of information and communication technology (ICT) such as internets, computers, smartphones, tablets, electronic wearables, and monitoring devices which enable digital interventions tailored to individual needs^1^. Ranging from promoting the adoption of healthy behaviours^2^ to supporting patients’ self-management of chronic diseases and long-term health conditions^3^, digital communication through eHealth applications allows patients and healthcare providers to liaise and exchange crucial information for monitoring their physical and mental well-being, as well as informing healthcare decisions^4–8^. Previous literature has suggested that mobile applications had a positive impact on diabetes self-management, as analysis indicated an association between application usage and improved attitudes favourable to diabetes self-management^9^. Also, statistically significant improvement in HbA1c level, diabetes knowledge, and self-care behaviours was also observed^9,10^.

The Food and Health Bureau (FHB) and the Hospital Authority (HA) of Hong Kong has developed an electronic Health Record Sharing System (eHRSS) and an eHealth Application. The eHRSS aimed to provide a free and lifelong electronic health records for the general population by enabling a two-way sharing system among public and private healthcare providers for healthcare purposes in the presence of patient consent. The eHealth application (“The app”) is an innovative tool that leverages the advantages of the eHRSS, which allows its downloading and adoption by the general public. Major functions of the app included the provision of general public health information and news, the viewing of the users’ health records (medications, allergies, vaccine records, etc.), and management of eHealth accounts for users’ children aged under 16, etc. The subsequent introduction of the Health Management Module allows patients to record their blood pressure, blood sugar, and heart rate in the app to monitor the change in their health conditions. A reminder function is set up to alert users in regular measurement of their health indices. It is expected that the eHealth App and Health Management Module will enhance patient access to their own medical information, enabling personal reference or adoption by their caregivers or family members. This study aims to compare the control of HbA1c among: (a) non-users of the eHealth App; (b) those who used the eHealth App only; and (c) users of the eHealth App in conjunction with the Health Management Module among patients with type 2 diabetes. In addition, we identify the difference in the magnitude of the intervention effect among patients of different ages and sex, and evaluate the sociodemographic factors associated with the use of intervention.

This study shows that the adoption of eHealth management module and eHealth App usage is associated with more optimal HbA1c outcomes, with the strongest effect observed in the younger female subgroup. Higher proportion of optimal HbA1c level is also associated with older age, shorter history of diabetes, tertiary education, unemployment, non-smokers, higher-level physical activity, presence of hypertension, presence of chronic kidney disease, absence of dyslipidaemia, as well as the use and non-use of specific treatments (insulin, oral hypoglycaemic agents, lipid-lowering agents, and renal dialysis).

## Results

### Participant characteristics

We identified 122,548 eligible patients with type II diabetes who have provided consent to the HA for data sharing. They had received an annual assessment for diabetes complications in the public sector within Jan 2021 and May 2022. Among them, 109,823 were eligible for the study, as 10,611 did not have records on their HbA1c level, and 2114 participants had type 1 Diabetes and were hence excluded (Supplementary Fig. 1). On average, the data used were 6.09 months after the use of the App (SD: 3.71 months). The included participants had a mean age of 67.04 years (standard deviation (SD): 10.61) with diabetes diagnosed for a mean duration of 11.03 years (SD = 8.31). Among them, the majority were male (*n* = 55,956, 51.0%); secondary school graduates (*n* = 54,842, 49.9%); retired (*n* = 33,775, 30.8%); non-smokers (*n* = 79,009, 71.9%); non-drinkers (*n* = 77,011, 70.1%); and did not perform any physical activity of moderate intensity (*n* = 67,317, 61.3%). The most prevalent chronic condition reported among these participants was dyslipidaemia (*N* = 98,353, 89.6%), followed by hypertension (*N* = 83,347, 75.9%), central obesity (*N* = 78,622, 71.6%) and chronic kidney disease (*N* = 69,131, 62.9%). In terms of medications, 88.0% of the respondents (*N* = 96,656) were on oral hypoglycemic agents, 72.5% (*N* = 79,608) were on lipid-lowering medications, while 72.0% (*N* = 79,127) were taking antihypertensive drugs.

The study participants were divided into three groups according to their usage of the eHealth App and the eHealth management module. There were 76,356 participants who were non-users, 31,723 used eHealth App, and 1744 used the eHealth management module in addition to eHealth App. Among the three groups, participants who were non-users had the highest mean age (68.84 years, SD = 10.27) and were diagnosed for the longest mean duration (11.54 years, SD = 8.47); while the eHealth management module group was the youngest with a mean age of 60.70 (SD = 9.53) and the shortest mean duration of DM for 8.78 years (SD = 7.60). The non-users had the greatest proportion of females (52.9%, vs 40.7% in the eHealth app group and 30.7% in the eHealth management module group) and the lowest proportion of participants who achieved secondary educational level (54.4%, vs 74.6% in eHealth app group and 87.4% in the eHealth management group). In terms of chronic conditions and medications, there were small disparities among the three groups, with the non-user group having a higher prevalence in most of the conditions and medication prescriptions. The detailed participant characteristics were shown in Table 1.

**Table 1:** Characteristics of the participants.

	eHRSS only (*n*, %)	eHealth App (*n*, %)	eHealth management module (*n*, %)	Total (*n*, %)
Total	76356	31723	1744	109823
Age (years)				
<60	14656 (19.2%)	11642 (36.7%)	822 (47.1%)	27120 (24.7%)
60 or above	61700 (80.8%)	20081 (63.3%)	922 (52.9%)	82703 (75.3%)
Sex				
Male	35929 (47.1%)	18818 (59.3%)	1209 (69.3%)	55956 (51.0%)
Female	40427 (52.9%)	12905 (40.7%)	535 (30.7%)	53867 (49.0%)
Education				
No formal education	6958 (9.1%)	903 (2.8%)	20 (1.1%)	7881 (7.2%)
Primary	27508 (36.0%)	7001 (22.1%)	189 (10.8%)	34698 (31.6%)
Secondary	35441 (46.4%)	18298 (57.7%)	1103 (63.2%)	54842 (49.9%)
Tertiary	6111 (8.0%)	5366 (16.9%)	422 (24.2%)	11899 (10.8%)
Occupation				
Manual	9533 (12.5%)	5224 (16.5%)	256 (14.7%)	15013 (13.7%)
Non-manual	7849 (10.3%)	6426 (20.3%)	472 (27.1%)	14747 (13.4%)
Housemaker	20240 (26.5%)	5099 (16.1%)	160 (9.2%)	25499 (23.2%)
Retired	25358 (33.2%)	7986 (25.2%)	431 (24.7%)	33775 (30.8%)
Unemployed	1879 (2.5%)	922 (2.9%)	38 (2.2%)	2839 (2.6%)
Others	5708 (7.5%)	3544 (11.2%)	241 (13.8%)	9493 (8.6%)
Smoking status				
Non-smoker	55586 (72.8%)	22208 (70.0%)	1215 (69.7%)	79009 (71.9%)
Current smoker	6753 (8.8%)	3567 (11.2%)	159 (9.1%)	10479 (9.5%)
Ex-smoker	13913 (18.2%)	5911 (18.6%)	368 (21.1%)	20192 (18.4%)
Alcohol				
Non-drinker	55050 (72.1%)	20868 (65.8%)	1093 (62.7%)	77011 (70.1%)
Current drinker	1780 (2.3%)	949 (3.0%)	61 (3.5%)	2790 (2.5%)
Social drinker	9492 (12.4%)	6027 (19.0%)	395 (22.6%)	15914 (14.5%)
Ex-drinker	3982 (5.2%)	1417 (4.5%)	83 (4.8%)	5482 (5.0%)
Physical activity of moderate intensity				
None	47862 (62.7%)	18534 (58.4%)	921 (52.8%)	67317 (61.3%)
<150 min per week	6822 (8.9%)	3462 (10.9%)	236 (13.5%)	10520 (9.6%)
At least 150 min per week	15809 (20.7%)	7243 (22.8%)	425 (24.4%)	23477 (21.4%)
Complications				
Central obesity				
No	18970 (24.8%)	8747 (27.6%)	496 (28.4%)	28213 (25.7%)
Yes	55162 (72.2%)	22250 (70.1%)	1210 (69.4%)	78622 (71.6%)
Hypertension				
No	16897 (22.1%)	9086 (28.6%)	493(28.3%)	26476 (24.1%)
Yes	59459 (77.9%)	22637 (71.4%)	1251 (71.7%)	83347 (75.9%)
Dyslipidaemia				
No	5091 (6.7%)	2182 (6.9%)	122 (7.0%)	7395 (6.7%)
Yes	68402 (89.6%)	28376 (89.4%)	1575 (90.3%)	98353 (89.6%)
Stroke				
No	69303 (90.8%)	29974 (94.5%)	1658 (95.1%)	100935 (91.9%)
Yes	6679 (8.7%)	1588 (5.0%)	74 (4.2%)	8341 (7.6%)
Coronary Heart Disease				
No	65967 (86.4%)	27810 (87.7%)	1480 (84.9%)	95257 (86.7%)
Yes	9979 (13.1%)	3721 (11.7%)	251 (14.4%)	13951 (12.7%)
Peripheral Arterial Disease				
No	68245 (89.4%)	28585 (90.1%)	1600 (91.7%)	98430 (89.6%)
Yes	622 (0.8%)	132 (0.4%)	6 (0.3%)	760 (0.7%)
Chronic Kidney disease				
No	20659 (27.1%)	11960 (37.7%)	685 (39.3%)	33304 (30.3%)
Yes	50966 (66.7%)	17249 (54.4%)	916 (52.5%)	69131 (62.9%)
Malignancy				
No	61433 (80.5%)	26346 (83.1%)	1483 (85.0%)	89262 (81.3%)
Yes	6640 (8.7%)	1795 (5.7%)	89 (5.1%)	8524 (7.8%)
Diabetic Retinopathy				
No	50516 (66.2%)	23138 (72.9%)	1330 (76.3%)	74984 (68.3%)
Yes	2962 (3.9%)	1042 (3.3%)	53 (3.0%)	4057 (3.7%)
Medications				
Insulin treatment				
No	61239 (80.2%)	26121 (82.3%)	1478 (84.7%)	88838 (80.9%)
Yes	9130 (12.0%)	3192 (10.1%)	165 (9.5%)	12487 (11.4%)
Antidiabetic drug				
No	8979 (11.8%)	3696 (11.7%)	251 (14.4%)	12926 (11.8%)
Yes	67185 (88.0%)	27981 (88.2%)	1490 (85.4%)	96656 (88.0%)
Antihypertensive drug				
No	14475 (19.0%)	7814 (24.6%)	422 (24.2%)	22711 (20.7%)
Yes	56260 (73.7%)	21645 (68.2%)	1222 (70.1%)	79127 (72.0%)
Lipid-lowering drug				
No	14971 (19.6%)	6847 (21.6%)	413 (23.7%)	22231 (20.2%)
Yes	55771 (73.0%)	22606 (71.3%)	1231 (70.6%)	79608 (72.5%)
Antiplatelet drug				
No	57058 (74.7%)	25283 (79.7%)	1347 (77.2%)	83688 (76.2%)
Yes	18954 (24.8%)	6329 (20.0%)	388 (22.2%)	25671 (23.4%)
Dialysis				
No	75737 (99.2%)	31502 (99.3%)	1731 (99.3%)	108970 (99.2%)
Yes	270 (0.4%)	60 (0.2%)	1 (0.1%)	331 (0.3%)
Duration of Diabetes				
< 10 years	39950 (52.3%)	19157 (60.4%)	1146 (65.7%)	60253 (54.9%)
10 years or above	36188 (47.4%)	12497 (39.4%)	596 (34.2%)	49281 (44.9%)

### Factors associated with optimal HbA1c level

First and foremost, the use of eHealth management module (aOR = 1.40, 95% CI = 1.26–1.56, *p* < 0.001) and eHealth app (aOR = 1.11, 95% CI = 1.08–1.15, *p* < 0.001) had significantly positive associations with optimal glycaemic control, with the effects being more evident among the eHealth management module group.

Several demographic and lifestyle factors were found to be associated with an optimal level of HbA1c (Tables 2 and 3). It was found that people aged over 60 were significantly more likely to achieve optimal levels (56.2% (proportion of participants who achieved optimal levels), vs 53.3% (aged below 60 who have achieved optimal levels); aOR = 1.12, 95% CI = 1.08–1.16, *p* < 0.001). Longer duration of more than 10 years since the diabetes diagnosis was negatively associated with optimal HbA1c level (45.5% vs 63.7%; aOR = 0.63, 95% CI = 0.62–0.65, *p* < 0.001). Compared to people who reported no formal education, participants with tertiary education had a higher odds of optimal glycaemic control (57.4% vs 55.8%; aOR = 1.15, 95% CI = 1.08–1.23, *p* < 0.001). Occupation was a significant factor associated with optimal HbA1c level, as participants who were housewives (58.1%; aOR = 1.25, 95% CI = 1.19–1.32, *p* < 0.001), retired (57.3%; aOR = 1.20, 95% CI = 1.15–1.25), and unemployed (53.7%; aOR = 1.19, 95% CI = 1.09–1.30, *p* < 0.001) had significantly better HbA1c level than manual workers (53.8%). Smoking was associated with a high HbA1c level, as current smokers (50.4%; aOR = 0.81, 95% CI = 0.78–0.85, *p* < 0.001) and ex-smokers (52.8%; aOR = 0.92, 95% CI = 0.89–0.96, *p* < 0.001) had significantly poorer control than non-smokers (56.8%). At least 150 min of weekly physical activity of moderate intensity was a protective factor (59.6%; aOR = 1.06, 95% CI = 1.03–1.10, *p* < 0.001) compared to participants who were physically inactive (54.8%).

**Table 2:** Factors associated with optimal control of HbA1c in all participants.

	*n*	Prevalence (%)	Crude odd ratio (cOR) (95% CI)	*p* value	Adjusted odd ratio (aOR) (95% CI)^a^	*p* value
Group						
eHRSS only	41,675	54.6	Ref		Ref	
eHealth app	18,159	57.2	1.11 (1.09–1.14)	<0.001*	1.11 (1.08–1.15)	<0.001*
Module adopted	1101	63.5	1.43 (1.29–1.57)	<0.001*	1.40 (1.26–1.56)	<0.001*

^a^adjusted for age group, sex, education, occupation, smoking status, alcohol drinking habit, physical activity of moderate intensity, presence of complications (central obesity, hypertension, dyslipidaemia, stroke, coronary heart disease, peripheral arterial disease, chronic kidney disease, malignancy, and diabetic retinopathy), use of medications (insulin treatment, antidiabetic drug, antihypertensive drug, lipid-lowering drug, antiplatelet drug, and dialysis), and duration of diabetes [Binary logistic regression].

*statistically significant.

**Table 3:** Factors associated with optimal control of HbA1c in males aged 60 years and older.

	Aged below 60						Aged 60 or above					
	*n*	Prevalence (%)	Crude odd ratio (cOR) (95% CI)	*P* value	Adjusted odd ratio (aOR)^a^ (95% CI)	*P* value	*n*	Prevalence (%)	Crude odd ratio (cOR) (95% CI)	*P* value	Adjusted odd ratio (aOR)^a^ (95% CI)	*P* value
Group												
eHRSS only	3701	51.3	Ref		Ref		15567	54.2	Ref		Ref	
eHealth app	3792	52.9	1.06 (1.00–1.14)	0.068	1.05 (0.98–1.13)	0.178	6762	58.1	1.17 (1.12–1.22)	<0.001*	1.11 (1.06–1.16)	<0.001*
Module adopted	319	58.5	1.34 (1.12–1.60)	0.001*	1.33 (1.10–1.61)	0.003*	417	62.8	1.43 (1.22–1.67)	<0.001*	1.31 (1.10–1.55)	0.002*

^a^adjusted for education, occupation, smoking status, alcohol drinking habit, physical activity of moderate intensity, presence of complications (central obesity, hypertension, dyslipidaemia, stroke, coronary heart disease, peripheral arterial disease, chronic kidney disease, malignancy, and diabetic retinopathy), use of medications (insulin treatment, antidiabetic drug, antihypertensive drug, lipid-lowering drug, antiplatelet drug, and dialysis), and duration of diabetes [Binary logistic regression].

*statistically significant.

Some chronic conditions were associated with worse glycaemic control, namely dyslipidaemia (54.6% vs 63.1% in participants without the condition; aOR = 0.63, 95% CI = 0.59–0.67, *p* < 0.001), diabetic retinopathy (37.9% vs 53.2%; aOR = 0.66, 95% CI = 0.61–0.70, *p* < 0.001) and central obesity (53.7% vs 61.1%; aOR = 0.71, 95% CI = 0.69–0.74, *p* < 0.001). On the contrary, patients with hypertension (55.6% vs 55.2%; aOR = 1.08, 95% CI = 1.05–1.12, *p* < 0.001) and chronic kidney disease (55.5% vs 55.3%; aOR = 1.08, 95% CI = 1.05–1.12, *p* < 0.001) were associated with better glycaemic control.

As far as the association between HbA1c level and medication is concerned, participants who were taking oral hypoglycaemic agents (51.7% vs 83.8% in participants who were not taking medications; aOR = 0.27, 95% CI = 0.25–0.28, *p* < 0.001), antiplatelet agents (51.4% vs 56.7%; aOR = 0.94, 95% CI = 0.90–0.98, *p* = 0.005), and insulin treatment (23.2% vs 59.5%; aOR = 0.28, 95% CI = 0.27–0.30, *p* < 0.001) had significantly worse glycaemic control. In contrast, patients who were taking lipid-lowering agents (54.6% vs 56.5%; aOR = 1.24, 95% CI = 1.19–1.29, *p* < 0.001) and had undergone renal dialysis (53.2% vs 55.5%; aOR = 2.25, 95% CI = 1.77–2.85, *p* < 0.001) were associated with better glycaemic control.

### Factors associated with optimal HbA1c level—subgroup analysis

Among younger males aged below 60, higher proportion of optimal HbA1c level was associated with the use of the Health Management Module (aOR = 1.33, 95% CI = 1.10–1.61); having retired (aOR = 1.40, 1.15–1.69) or being unemployed (aOR = 1.22, 95% CI = 1.07–1.40) (compared to manual workers); having at least 150 min of physical activities of moderate intensity (aOR = 1.21, 95% CI = 1.10–1.32); having chronic kidney disease (aOR = 1.10, 95% CI = 1.02–1.18); the use of lipid-lowering drug (aOR = 1.24, 95% CI = 1.13–1.36); and having undergone renal dialysis (aOR = 2.45, 95% CI = 1.45–4.13). On the contrary, lower proportion of optimal HbA1c level was associated with current cigarette smoking (aOR = 0.82, 95% CI = 0.75–0.89, compared with non-smoker); the presence of central obesity (aOR = 0.70, 95% CI = 0.64–0.75), dyslipidaemia (aOR = 0.58, 95% CI = 0.50, 0.67), diabetic retinopathy (aOR = 0.71, 95% CI = 0.60–0.85); use of insulin therapy (aOR = 0.33, 95% CI = 0.29–0.37), oral hypoglycemic drugs (aOR = 0.22, 95% CI = 0.19–0.26), and diabetes duration of DM for over 10 years (aOR = 0.62, 95% CI = 0.57–0.67). On the other hand, for older males aged 60 or above, both the use of the App (aOR = 1.11, 95% CI = 1.06–1.16) and the Module (aOR = 1.31, 95% CI = 1.10–1.55) were significantly associated with optimal glycaemic control. Similarly, optimal level of HbA1c was associated with other factors including occupational status, smoking status (current or ex-), presence of certain complications, the use of certain medications, and longer duration of diabetes. The detailed results of the regression analysis among male patients are listed in Table 3.

Among younger females aged below 60, optimal HbA1c control was positively associated with the use of the app (aOR = 1.17, 95% CI = 1.08–1.26) and the Health Management Module (aOR = 1.66, 95% CI = 1.27–2.17); occupation as housewives (aOR = 1.20, 95% CI = 1.07–1.35); having coronary heart disease (aOR = 1.47, 95% CI = 1.12–1.92), chronic kidney disease (aOR = 1.21, 95% CI = 1.10–1.33); the use of lipid-lowering drug (aOR = 1.24, 95% CI = 1.13–1.37), and renal dialysis (aOR = 5.21, 95% CI = 2.04–13.26). Current smoking (aOR = 0.78, 95% CI = 0.66–0.91), social drinking (aOR = 0.87, 95% CI = 0.77–0.98), the presence of central obesity (aOR = 0.72, 95% CI = 0.64–0.80), dyslipidaemia (aOR = 0.61, 95% CI = 0.52–0.72), diabetic retinopathy (aOR = 0.51, 95% CI = 0.40–0.65), the use of insulin treatment (aOR = 0.32, 95% CI = 0.28–0.37), and oral hypoglycaemic agents (aOR = 0.24, 95% CI = 0.21–0.28), and longer duration of diabetes (aOR = 0.69, 95% CI = 0.63–0.76). Likewise, optimal HbA1c control was positively associated with both the use of the app (aOR = 1.13, 95% CI = 1.08–1.20) and the module (aOR = 1.50, 95% CI = 1.13–1.99). The associations between optimal HbA1c level and other factors were similar in their younger counterparts. The full set of findings from regression analysis among female individuals can be found in Table 4.

**Table 4:** Factors associated with optimal control of HbA1c in females aged 60 years and older.

	Aged below 60						Aged 60 or above					
	*n*	Prevalence (%)	Crude odd ratio (cOR) (95% CI)	*P* value	Adjusted odd ratio (aOR)^a^ (95% CI)	*P* value	*n*	Prevalence (%)	Crude odd ratio (cOR) (95% CI)	*P* value	Adjusted odd ratio (aOR)^a^ (95% CI)	*P* value
Group												
eHRSS only	3923	52.7	Ref		Ref		18,484	56.0	Ref		Ref	
eHealth app	2524	56.5	1.17 (1.08–1.26)	<0.001*	1.17 (1.08–1.26)	<0.001*	5081	60.2	1.19 (1.13–1.25)	<0.001*	1.13 (1.08–1.20)	<0.001*
Module adopted	184	66.4	1.78 (1.38–2.29)	<0.001*	1.66 (1.27–2.17)	<0.001*	181	70.2	1.84 (1.41–2.41)	<0.001*	1.50 (1.13–1.99)	0.005*

^a^adjusted for education, occupation, smoking status, alcohol drinking habit, physical activity of moderate intensity, presence of complications (central obesity, hypertension, dyslipidaemia, stroke, coronary heart disease, peripheral arterial disease, chronic kidney disease, malignancy, and diabetic retinopathy), use of medications (insulin treatment, antidiabetic drug, antihypertensive drug, lipid-lowering drug, antiplatelet drug, and dialysis), and duration of diabetes [Binary logistic regression].

*statistically significant.

### Factors associated with the use of the App and the Health Management Module

Several factors were associated with the use of the App (Table 5). Compared to patients aged below 29, patients who were 50–64 (aOR = 0.66, 95% CI = 0.50–0.87), 65–79 (aOR = 0.41, 95% CI:0.31–0.55), and 80 or above (aOR = 0.18, 95% CI = 0.14–0.25) were significantly less likely to have used the App. Female was evidently less likely to have used the app (aOR = 0.73, 95% CI: 0.71–0.76). Higher education level was positively associated with the likelihood of using the app (aOR_primary_ = 1.41, 95% CI = 1.31–1.52; aOR_secondary_ = 2.15, 95% CI = 2.00–2.32; aOR_tertiary_ = 3.23, 95% CI = 2.97–3.51, compared to patients with no formal education). Compared to manual workers, non-manual workers (aOR = 1.24, 95% CI = 1.18–1.30) were more likely to have used the app, whilst housemakers (aOR = 0.86. 95% CI = 0.82–0.91), those who have retired (aOR = 0.88, 95% CI: 0.84–0.92), and patients who were unemployed (aOR = 0.76, 95% CI = 0.69–0.83) were less likely to have used the app. Compared to non-smokers, current smokers (aOR = 0.87, 95% CI: 0.83–0.91) and ex-smokers (aOR = 0.92, 95% CI: 0.88–0.95) were less likely to have used the app. In terms of drinking habits, current drinkers (aOR = 1.17, 95% CI: 1.08–1.28) and social drinkers (aOR = 1.24, 95% CI: 1.19–1.29) were more likely to have used the app, while ex-drinkers (aOR = 0.86, 95% CI: 0.81–0.92) were less likely to have used the app. Meanwhile, patients who have done physical activities of moderate intensity for less than 150 min (aOR = 1.12, 95% CI: 1.07–1.18) or at least 150 min (aOR = 1.15, 95% CI: 1.11–1.19) per week were significantly more likely to have used the app compared to patients who have not performed any physical activities.

**Table 5:** Factors associated with the usage of eHealth app.

	*n*	Prevalence (%)	Crude odd ratio (cOR) (95% CI)	*P* value	Adjusted odd ratio (aOR) 95% CI	*P* value
Age (years)						
Below 29	115	56.1	Ref		Ref	
30–39	627	58.3	1.09 (0.81–1.48)	0.563	1.09 (0.81–1.49)	0.566
40–49	2543	52.7	0.87 (0.66–1.15)	0.336	0.92 (0.69–1.23)	0.574
50–64	14730	40.8	0.54 (0.41–0.71)	<0.001*	0.66 (0.50–0.87)	<0.001*
65–79	14097	25.4	0.27 (0.20–0.35)	<0.001*	0.41 (0.31–0.55)	<0.001*
80 or above	1355	11.2	0.10 (0.08–0.13)	<0.001*	0.18 (0.14–0.25)	<0.001*
Sex						
Male	20027	35.8	Ref		Ref	
Female	13440	25.0	0.60 (0.58–0.61)	<0.001*	0.73 (0.71–0.76)	<0.001*
Education						
No formal education	923	11.7	Ref		Ref	
Primary	7190	20.7	1.97 (1.83–2.12)	<0.001*	1.41 (1.31–1.52)	<0.001*
Secondary	19401	35.4	4.13 (3.84–4.43)	<0.001*	2.15 (2.00–2.32)	<0.001*
Tertiary	5788	48.6	7.14 (6.61–7.72)	<0.001*	3.23 (2.97–3.51)	<0.001*
Occupation						
Manual	5480	36.5	Ref		Ref	
Non-manual	6898	46.8	1.53 (1.46–1.60)	<0.001*	1.24 (1.18–1.30)	<0.001*
Housemaker	5259	20.6	0.45 (0.43–0.47)	<0.001*	0.86 (0.82–0.91)	<0.001*
Retired	8417	24.9	0.58 (0.55–0.60)	<0.001*	0.88 (0.84–0.92)	<0.001*
Unemployed	960	33.8	0.89 (0.82–0.97)	0.006*	0.76 (0.69–0.83)	<0.001*
Others	3785	39.9	1.15 (1.09–1.22)	<0.001*	1.06 (1.01–1.12)	0.029*
Smoking status						
Non-smoker	23423	29.6	Ref		Ref	
Current smoker	3726	35.6	1.31 (1.26–1.37)	<0.001*	0.87 (0.83–0.91)	<0.001*
Ex-smoker	6279	31.1	1.07 (1.04–1.11)	<0.001*	0.92 (0.88–0.95)	<0.001*
Alcohol						
Non-drinker	21961	28.5	Ref		Ref	
Current drinker	1010	36.2	1.42 (1.32–1.54)	<0.001*	1.17 (1.08–1.28)	<0.001*
Social drinker	6422	40.4	1.70 (1.64–1.76)	<0.001*	1.24 (1.19–1.29)	<0.001*
Ex-drinker	1500	27.4	0.94 (0.89–1.00)	0.067	0.86 (0.81–0.92)	<0.001*
Physical activity of moderate intensity						
None	19455	28.9	Ref		Ref	
< 150 min per week	3698	35.2	1.33 (1.28–1.39)	<0.001*	1.12 (1.07–1.18)	<0.001*
At least 150 min per week	7668	32.7	1.19 (1.16–1.23)	<0.001*	1.15 (1.11–1.19)	<0.001*

*statistically significant.

Among patients who used the app, significantly higher adoption rate of the Health Management Module was associated with higher education level (aOR_secondary_ = 1.77, 95% CI = 1.12–2.79; aOR_tertiary_ = 2.03, 95% CI = 1.27–3.25, compared to patients with no formal education), non-manual works (aOR = 1.30, 95% CI = 1.10–1.52), having retired (aOR = 1.29, 95% CI = 1.10–1.58), and engaging in physical activities of moderate intensity for less than 150 min per week (aOR = 1.23, 95% CI: 1.06–1.42, compared to patients who do not do any physical activities); while patients who were aged 80 or above (aOR = 0.49, 95% CI = 0.29–0.81), female individuals (aOR = 0.75, 95% CI = 0.66–0.85), and current smokers (aOR = 0.67, 95% CI: 0.56–0.60) had a lower rate of adopting the module (Table 6).

**Table 6:** Factors associated with the usage of Health Management Module among eHealth app users.

	*n*	Prevalence (%)	Crude odd ratio (cOR) (95% CI)	*P* value	Adjusted odd ratio (aOR) 95% CI	*P* value
Age (years)						
Below 29	1	0.9	0.14 (0.02–1.0003)	0.050	0.13 (0.02–0.96)	0.045*
30–39	38	6.1	Ref		Ref	
40–49	175	6.9	1.15 (0.80–1.65)	0.462	1.17 (0.81–1.68)	0.402
50–64	898	6.1	1.01 (0.72–1.41)	0.971	1.08 (0.77–1.51)	0.664
65–79	601	4.3	0.69 (0.49–0.97)	0.032*	0.80 (0.56–1.14)	0.218
80 or above	31	2.3	0.36 (0.22–0.59)	<0.001*	0.49 (0.29–0.81)	0.005*
Sex						
Male	1209	6.0	Ref		Ref	
Female	535	4.0	0.65 (0.58–0.72)	<0.001*	0.75 (0.66–0.85)	<0.001*
Education						
No formal education	20	2.2	Ref		Ref	
Primary	189	2.6	1.22 (0.77–1.94)	0.405	0.96 (0.60–1.53)	0.856
Secondary	1103	5.7	2.72 (1.74–4.26)	<0.001*	1.77 (1.12–2.79)	0.015*
Tertiary	422	7.3	3.55 (2.26–5.59)	<0.001*	2.03 (1.27–3.25)	0.003*
Occupation						
Manual	256	4.7	Ref		Ref	
Non-manual	472	6.8	1.50 (1.28–1.75)	<0.001*	1.30 (1.10–1.52)	0.002*
Housemaker	160	3.0	0.64 (0.52–0.78)	<0.001*	1.00 (0.80–1.25)	0.991
Retired	431	5.1	1.10 (0.94–1.29)	0.233	1.29 (1.10–1.58)	0.005*
Unemployed	38	4.0	0.84 (0.59–1.19)	0.329	0.76 (0.53–1.07)	0.118
Others	241	6.4	1.39 (1.16–1.66)	<0.001*	1.32 (1.10–1.58)	0.003*
Smoking status						
Non-smoker	1215	5.2	Ref		Ref	
Current smoker	159	4.3	0.82 (0.69–0.97)	0.018*	0.67 (0.56–0.80)	<0.001*
Ex-smoker	368	5.9	1.14 (1.01–1.28)	0.035*	1.00 (0.88–1.15)	0.957
Alcohol						
Non-drinker	1093	5.0	Ref		Ref	
Current drinker	61	6.0	1.23 (0.94–1.60)	0.131	1.17 (0.89–1.53)	0.273
Social drinker	395	6.2	1.25 (1.11–1.41)	<0.001*	1.06 (0.93–1.20)	0.381
Ex-drinker	83	5.5	1.12 (0.89–1.41)	0.340	1.03 (0.81–1.31)	0.835
Physical activity of moderate intensity						
None	921	4.7	Ref		Ref	
< 150 min per week	236	6.4	1.37 (1.18–1.59)	<0.001*	1.23 (1.06–1.42)	0.008*
At least 150 min per week	425	5.5	1.18 (1.05–1.33)	0.006*	1.10 (0.98–1.24)	0.116

*statistically significant.

## Discussion

Self-management of blood glucose levels is vital for patients with diabetes mellitus. The prevalence of type 2 diabetes is higher amongst middle-aged and older people, yet often those belonging to older age groups are more reluctant to engage with eHealth technologies. Majority of non-users from the current study were aged 60 or above, making up a large proportion of the non-user (80.8%) and eHealth group (63.3%) - whilst older users of the eHealth management module were around 52.9%. Although individuals have been encouraged to sign up for eHRSS to facilitate the sharing of health-related data between medical practitioners and patients, registration does not necessitate usage of the accompanying eHealth App. A review of past literature has determined barriers such as, technological and health illiteracy, low awareness of existing apps, not owning a device capable of accessing eHealth, and an absence of desire to change health behaviour may deter individuals from accessing digital solutions^11,12^. The approximately equal distribution of younger and older eHealth App management module users may be indicative of (1) lower adoption of technology among older adults, much less the use of digital media to assist self-management of health^13,14^; (2) younger adults taking initiative to improve their health status as they are one of the most active groups of internet users that search for online health-related information^15,16^. A higher level of educational attainment has been previously associated with higher eHealth literacy, increased usage of digital health technologies, and increased health consciousness^17^. A study in Taiwan found a greater level of mobile eHealth literacy in type 2 diabetes patients with higher levels of education, which suggests that mobile eHealth applications may serve to enhance health behaviours in the diabetes population^18^.

Diabetes management has relied on effective glycaemic control by monitoring the Haemoglobin A1c (HbA1c) value as it acts as an indicator of average blood glucose over a three-month period, with the optimal range being below 7.0%^19^. Poor glycaemic control has been associated with younger age, duration of diabetes, age of onset, family history, job status, educational status, anti-diabetic drugs used for treatment, and presence of hypertension^20^, which were generally consistent with our findings. Individuals who are 60 years and older have previously demonstrated better glycaemic control in a meta-analysis of ten studies, which is further supported by optimal HbA1c outcomes in the older participant group (≥60) in the overall regression analysis of our study. This may be attributed to increased awareness of taking care of one’s health as chronic conditions become more impactful with older age^21^. In addition, diabetes duration has been found to be an influential factor that may determine poorer or better HbA1c outcomes. Studies in India and Morocco found that significant increases in HbA1c and insulin levels were associated with longer duration of diabetes, which might be due to a gradual rise in insulin resistance^22,23^. Similar to the aforementioned effect of eHealth App usage in relation to educational status, a cross-sectional study in Malaysia reported significantly lower HbA1c values amongst those with a higher level of education (*p* < 0.05), which is compatible with better HbA1c outcomes found in the tertiary education subgroup of this study. Moreover, poorer glycaemic control has been found in participants who had only completed primary education in a Turkish study^20^ and poorer HbA1c trajectories were associated with a lower educational level in a review of twenty studies^24^. Better HbA1c outcomes associated with hypertension and chronic kidney disease in the current study contrasts with past studies as these health conditions have often been indicators of poorer glycaemic control with patients experiencing health complications such as deteriorating kidney function^20,24^, However, the associations were of a smaller magnitude than other factors, such as the use of the Health Management Module and medication. There might also be other unexplored confounders that led to the associations. Treatment options for diabetes may range from oral medication to insulin treatments. Mixed results were reported between HbA1c outcomes and insulin delivery via injection versus an insulin pump as a review of multiple studies found both significant and non-significant associations^24^. However, our findings demonstrated significantly worse outcomes in patients who were taking anti-diabetic medication, anti-platelet medication, or receiving insulin treatments. Those who engage in regular physical activity and are non-smokers have a better overall health status, thus it was unsurprising to observe better HbA1c outcomes in these individuals. Exercise has been shown to enhance better management of DM, as a sizeable reduction in HbA1c levels found in a meta-analysis and case-control intervention study^25,26^, whilst non-smokers or smoking cessation can lead to lower HbA1c levels^27,28^.

Use of eHealth mobile applications specifically targeting the management of diabetes through behavioural interventions and recommended lifestyle changes have been proven beneficial across the population. Females have reported higher use of eHealth apps compared to their male counterparts in the United States (29% vs 19%)^29^ whilst men in Korea have reported higher levels of health consciousness and eHealth literacy when compared with that of women^17^. Our finding regarding sex difference was in line with the Korean study, which may be suggestive of a higher level of eHealth literacy among males than females in Asia. However, younger females demonstrated the strongest effect in the association between HbA1c outcomes and usage of eHealth app and the module compared to other subgroups in this study, highlighting potential application of promoting the app and the module to the female population. Across majority of studies it is consistently suggested that younger age groups possess higher levels of eHealth and technological literacy which consequently leads to increased app usage^11–13,17,30^. Future studies may explore effective ways to promote the intervention among the elderly population.

Although the study had a relatively large sample size which may facilitate its generalisability to other settings, a few limitations should be addressed. Small difference may become statistically significant even if the association was not clinically significant due to the large sample size – and hence the findings should be cautiously interpreted. Owing to the cross-sectional nature of the study, a cause-and-effect relationship between eHealth usage and glycaemic control cannot be established due to possibility of reverse causality. Furthermore, there is a possibility of residual confounders despite the use of regression modelling.

A significant difference in clinical outcomes, observed through glycaemic control, was found between non-users and eHealth App users, particularly among eHealth Management Module users. Although better HbA1c outcomes are generally associated with older age, majority of app users tend to be younger adults. Certain socio-demographic factors, medical conditions, duration of DM, and medical treatments may affect overall HbA1c outcomes. Hence, further research should be conducted to explore the association between optimal HbA1c level and time spent on using the module.

## Methods

### Study setting

Patient data were retrieved from the Clinical Data Analysis and Reporting System (CDARS) of the Hong Kong Hospital Authority (HA), which contains health information of all public hospitals and clinical settings across various regions (i.e., the New Territories, Hong Kong Island, and Kowloon). These databases were used to compare the control of HbA1c between the 3 groups of patients with type 2 diabetes, which included (1) non-users of the eHealth App, (2) users of only the eHealth App, (3) users of the eHealth App with the Health Management Module. The present study was performed in accordance with the ethical guidelines of the Declaration of Helsinki. The study was approved by the Survey and Behavioural Research Ethics Committee of the Chinese University of Hong Kong (No. SBRE-21-0950). Written informed consent was waived as this was a retrospective database analysis without identifying information of participants.

### Eligible participants

Eligible participants were all patients in Hong Kong who (1) had type II diabetes mellitus (DM), (2) had received annual assessment for diabetes complications in the public sector during the period January 2021 and May 2022, (3) provided consent to share their medical record on the electronic Health Record Sharing System (eHRSS). We excluded patients who were diagnosed with type I diabetes and those who had no HbA1c records in the computer system. Eligible participants were divided into three groups based on their usage of the eHealth app and the health management module. The usage was defined by having accessed the eHealth app and the health management module, respectively.

### Data collection

The CDARS of the HA was accessed to retrieve health information including: (1) patient socio-demographics: age, sex, education level, and occupational status; (2) lifestyle habits, such as cigarette smoking, alcohol consumption, and physical activity; (3) clinical parameters including glycated haemoglobin levels, lipid profile, presence of diabetes complications, prescribed medications, and duration of diabetes diagnosis; (4) patient enrolment status in the eHRSS and the usage level of the eHealth App. The CDARS is an electronic healthcare database that consists of patient demographic data, disease diagnoses, clinical procedures, drug prescriptions and laboratory results from all public hospitals and clinics in Hong Kong. The data on comorbidities were coded by International Classification of Diseases Ninth Revision, Clinical Modification (ICD-9-CM) in CDARS, which have been validated in clinical, laboratory, imaging and endoscopy results from the electronic medical records. Sociodemographic data including the year of birth, sex, smoking status, alcohol consumption, physical examination results, relevant laboratory investigations and drug prescriptions were collected. Coexisting medical conditions in each patient were also extracted with the use of all relevant ICD-9-CM diagnosis and procedure codes. The accuracy of the territory-wide database has been validated, and it was found that it consists of complete (100%) patient demographic variables^31^.

### Statistical analysis

Data were entered into IBM Statistical Package for Social Sciences (SPSS) version 25 software to conduct statistical analyses. A descriptive analysis of study participants was performed based on their demographic details and socio-economic status. The association between desired glycated haemoglobin outcomes and non-usage of the eHealth App, eHealth App usage, and eHealth Management Module usage was examined. The level of optimal glycated haemoglobin (defined as <7%) was the outcome variable whilst all other factors were considered as explanatory variables. Separate binary logistic models were constructed to examine the factors listed above and derive the crude odd ratio (cOR). The variables were tested for interaction and multi-collinearity whilst multiple logistic regression analysis was used to examine the associations. We controlled for potential confounding variables and computed the respective adjusted odd ratios (aORs). Similarly, additional subgroup analyses were conducted using multiple logistic regression to examine the association for four subgroups: males aged below 60, females aged below 60, males aged 60 or above, and females aged 60 or above. Furthermore, multiple logistic regression was conducted to evaluate factors associated with the adoption of the eHealth app and the Health Management Module. All *p* values less than 0.05 were considered statistically significant multivariate regression analysis.

### Reporting summary

Further information on research design is available in the Nature Research Reporting Summary linked to this article.

## Supplementary information

Supplementary info

REPORTING SUMMARY

## Data Availability

The data that support the findings of this study are available from the corresponding author upon reasonable request.
