# Supplementary material for: Associations between adoption of eHealth management module and optimal control of HbA1c in diabetes patients
Source: NPJ Digit Med. 2023 Apr 13;6:67. doi: 10.1038/s41746-023-00807-w (PMC10101956; doi:10.1038/s41746-023-00807-w)

## Supplementary Item legends

**Supplementary table 1.** Factors associated with optimal control of HbA1c in all participants [Binary logistic regression]

**Supplementary table 2.** Factors associated with optimal control of HbA1c in males [Binary logistic regression]

**Supplementary table 3.** Factors associated with optimal control of HbA1c in females [Binary logistic regression]

**Supplementary figure 1.** Flow chart for participant selection and categorization

**Supplementary table 1.** Factors associated with optimal control of HbA1c in all participants

| | n | Prevalence (%) | Crude odd ratio (cOR) (95% CI) | p-value | Adjusted odd ratio (aOR) (95% CI) | p-value |
|---|---|---|---|---|---|---|
| Age (years) | | | | | | |
| < 60 | 14443 | 53.3 | Ref | | Ref | |
| 60 or above | 46492 | 56.2 | 1.12 (1.09-1.15) | <0.001* | 1.12 (1.08-1.16) | <0.001* |
| Sex | | | | | | |
| Male | 30558 | 54.6 | Ref | | Ref | |
| Female | 30377 | 56.4 | 1.07 (1.05-1.10) | <0.001* | 1.03 (0.99-1.06) | 0.086 |
| Group | | | | | | |
| eHRSS only | 41675 | 54.6 | Ref | | Ref | |
| eHealth app | 18159 | 57.2 | 1.11 (1.08-1.14) | <0.001* | 1.11 (1.08-1.14) | <0.001* |
| Module adopted | 1101 | 63.5 | 1.42 (1.29-1.57) | <0.001* | 1.40 (1.26-1.55) | <0.001* |
| Education | | | | | | |
| No formal education | 4394 | 55.8 | Ref | | Ref | |
| Primary | 19204 | 55.3 | 0.98 (0.93-1.03) | 0.510 | 0.98 (0.93-1.03) | 0.510 |
| Secondary | 30180 | 55.0 | 0.97 (0.92-1.01) | 0.227 | 1.00 (0.95-1.06) | 0.792 |
| Tertiary | 6835 | 57.4 | 1.07 (1.01-1.13) | 0.019* | 1.15 (1.08-1.23) | <0.001* |
| Occupation | | | | | | |
| Manual | 8071 | 53.8 | Ref | | Ref | |
| Non-manual | 7903 | 53.6 | 0.99 (0.94-1.04) | 0.769 | 1.02 (0.97-1.07) | 0.319 |
| Housemaker | 14823 | 58.1 | 1.19 (1.14-1.24) | <0.001* | 1.25 (1.19-1.31) | <0.001* |
| Retired | 19344 | 57.3 | 1.15 (1.10-1.19) | <0.001* | 1.20 (1.14-1.25) | <0.001* |
| Unemployed | 1524 | 53.7 | 0.99 (0.92-1.08) | 0.938 | 1.19 (1.09-1.29) | <0.001* |
| Others | 5344 | 56.3 | 1.10 (1.05-1.16) | <0.001* | 1.13 (1.07-1.20) | <0.001* |
| Smoking status | | | | | | |
| Non-smoker | 44910 | 56.8 | Ref | | Ref | |
| Current smoker | 5281 | 50.4 | 0.85 (0.61-1.19) | 0.360 | 0.81 (0.77-0.85) | <0.001* |
| Ex-smoker | 10663 | 52.8 | 1.00 (0.72-1.40) | 0.962 | 0.92 (0.88-0.95) | <0.001* |
| Alcohol | | | | | | |
| Non-drinker | 42998 | 55.8 | Ref | | Ref | |
| Current drinker | 1598 | 57.3 | 1.06 (0.98-1.14) | 0.132 | 1.05 (0.96-1.14) | 0.250 |
| Social drinker | 8466 | 53.2 | 0.89 (0.86-0.93) | <0.001* | 0.97 (0.94-1.01) | 0.244 |
| Ex-drinker | 2707 | 49.4 | 0.77 (0.73-0.81) | <0.001* | 0.97 (0.92-1.04) | 0.495 |
| Physical activity of | | | | | | |
| None | 36865 | 54.8 | Ref | | Ref | |
| < 150 mins per | 6277 | 59.7 | 1.22 (1.17-1.27) | <0.001* | 0.96 (0.91-1.00) | 0.091 |
| At least 150 mins per week | 13991 | 59.6 | 1.21 (1.18-1.25) | <0.001* | 1.06 (1.02-1.09) | <0.001* |
| Complications | | | | | | |
| Central obesity | | | | | | |
| No | 17243 | 61.1 | Ref | | Ref | |
| Yes | 42193 | 53.7 | 0.73 (0.71-0.75) | <0.001* | 0.71 (0.69-0.73) | <0.001* |
| Hypertension | | | | | | |
| No | 14613 | 55.2 | Ref | | Ref | |
| Yes | 46322 | 55.6 | 1.01 (0.98-1.04) | 0.274 | 1.12 (1.07-1.18) | <0.001* |
| Dyslipidaemia | | | | | | |
| No | 4530 | 61.3 | Ref | | Ref | |
| Yes | 53731 | 54.6 | 0.76 (0.72-0.79) | <0.001* | 0.63 (0.59-0.67) | <0.001* |
| Stroke | | | | | | |
| No | 56160 | 55.6 | Ref | | Ref | |
| Yes | 4442 | 53.3 | 0.90 (0.86-0.95) | <0.001* | 1.00 (0.95-1.06) | 0.864 |

| | | | | | | |
|---|---|---|---|---|---|---|
| Coronary Heart Disease | | | | | | |
| No | 53344 | 6.0 | Ref | | Ref | |
| Yes | 7221 | 51.8 | 0.84 (0.81-0.87) | <0.001* | 0.99 (0.94-1.04) | 0.877 |
| Peripheral Arterial Disease | | | | | | |
| No | 54359 | 55.2 | Ref | | Ref | |
| Yes | 335 | 44.1 | 0.63 (0.55-0.73) | <0.001* | 0.98 (0.84-1.15) | 0.870 |
| Chronic Kidney disease | | | | | | |
| No | 18414 | 55.3 | Ref | | Ref | |
| Yes | 38396 | 55.5 | 1.01 (0.98-1.03) | 0.450 | 1.08 (1.04-1.11) | <0.001* |
| Malignancy | | | | | | |
| No | 49215 | 55.1 | Ref | | Ref | |
| Yes | 4711 | 55.3 | 1.00 (0.96-1.05) | 0.815 | 1.01 (0.96-1.06) | 0.638 |
| Diabetic Retinopathy | | | | | | |
| No | 42928 | 57.2 | Ref | | Ref | |
| Yes | 1537 | 37.9 | 0.45 (0.42-0.48) | <0.001* | 0.65 (0.61-0.70) | <0.001* |
| Medications | | | | | | |
| Insulin treatment | | | | | | |
| No | 52843 | 59.5 | Ref | | Ref | |
| Yes | 2896 | 23.2 | 0.20 (0.19-0.21) | <0.001* | 0.28 (0.27-0.29) | <0.001* |
| Antidiabetic drug | | | | | | |
| No | 10833 | 83.8 | Ref | | Ref | |
| Yes | 49939 | 51.7 | 0.20 (0.19-0.21) | <0.001* | 0.26 (0.25-0.27) | <0.001* |
| Antihypertensive drug | | | | | | |
| No | 12644 | 55.7 | Ref | | Ref | |
| Yes | 43415 | 54.9 | 0.96 (0.94-0.99) | 0.031* | 1.03 (0.98-1.08) | 0.185 |
| Lipid-lowering drug | | | | | | |
| No | 12569 | 56.5 | Ref | | Ref | |
| Yes | 43494 | 54.6 | 0.92 (0.89-0.95) | <0.001* | 1.24 (1.19-1.28) | <0.001* |
| Antiplatelet drug | | | | | | |
| No | 47456 | 56.7 | Ref | | Ref | |
| Yes | 13195 | 51.4 | 0.80 (0.78-0.83) | <0.001* | 0.93 (0.89-0.98) | 0.005* |
| Dialysis | | | | | | |
| No | 60435 | 55.5 | Ref | | Ref | |
| Yes | 176 | 53.2 | 0.91 (0.73-1.13) | 0.403 | 2.24 (1.77-2.85) | <0.001* |
| Duration of Diabetes | | | | | | |
| < 10 years | 38364 | 63.7 | Ref | | Ref | |
| 10 years or above | 22399 | 45.5 | 0.47 (0.46-0.48) | <0.001* | 0.63 (0.61-0.65) | <0.001* |

**Supplementary Table 2.** Factors associated with optimal control of HbA1c in males

| | Aged below 60 | | | | | | Aged 60 or above | | | | | |
|---|---|---|---|---|---|---|---|---|---|---|---|---|
| | n | Prevalence (%) | Crude odd ratio (cOR) (95% CI) | P value | Adjusted odd ratio (aOR) (95%CI) | P value | n | Prevalence (%) | Crude odd ratio (cOR) (95% CI) | P value | Adjusted odd ratio (aOR) (95% CI) | P value |
| Group | | | | | | | | | | | | |
| eHRSS only | 3701 | 51.3 | Ref | | Ref | | 15567 | 54.2 | Ref | | Ref | |
| eHealth app | 3792 | 52.9 | 1.06 (0.99-1.13) | 0.068 | 1.04 (0.97-1.12) | 0.178 | 6762 | 58.1 | 1.17 (1.12-1.22) | <0.001* | 1.10 (1.05-1.15) | <0.001* |
| Module adopted | 319 | 58.5 | 1.33 (1.12-1.59) | 0.001* | 1.33 (1.10-1.60) | 0.003* | 417 | 62.8 | 1.42 (1.21-1.67) | <0.001* | 1.30 (1.10-1.54) | 0.002* |
| Education | | | | | | | | | | | | |
| No formal education | 29 | 44.6 | Ref | | Ref | | 784 | 56.9 | Ref | | Ref | |
| Primary | 658 | 51.1 | 1.29 (0.78-2.14) | 0.310 | 1.21 (0.71-2.06) | 0.479 | 7088 | 54.1 | 0.89 (0.79-0.99) | 0.047* | 0.90 (0.80-1.01) | 0.091 |
| Secondary | 5192 | 51.9 | 1.33 (0.81-2.18) | 0.245 | 1.22 (0.72-2.06) | 0.452 | 11830 | 55.3 | 0.94 (0.84-1.04) | 0.272 | 0.94 (0.84-1.06) | 0.362 |
| Tertiary | 1877 | 54.2 | 1.46 (0.89-2.40) | 0.129 | 1.29 (0.76-2.19) | 0.332 | 2931 | 58.9 | 1.08 (0.96-1.22) | 0.177 | 1.13 (0.99-1.29) | 0.059 |
| Occupation | | | | | | | | | | | | |
| Manual | 2196 | 51.8 | Ref | | Ref | | 3003 | 54.7 | Ref | | Ref | |
| Non-manual | 2469 | 52.6 | 1.03(0.94-1.11) | 0.487 | 1.01 (0.92-1.11) | 0.748 | 2507 | 53.6 | 0.95 (0.88-1.03) | 0.269 | 1.01 (0.92-1.09) | 0.826 |
| Housemaker | 5 | 45.5 | 0.77 (0.23-2.54) | 0.673 | 0.76 (0.20-2.77) | 0.681 | 17 | 58.6 | 1.17 (0.55-2.46) | 0.673 | 1.27 (0.58-2.79) | 0.543 |
| Retired | 347 | 59.3 | 1.35 (1.13-1.61) | 0.001* | 1.39 (1.15-1.68) | 0.001* | 13114 | 57.1 | 1.10 (1.03-1.16) | 0.001* | 1.21 (1.13-1.29) | <0.001* |
| Unemployed | 656 | 54.2 | 1.10 (0.96-1.25) | 0.145 | 1.22 (1.06-1.40) | 0.004* | 429 | 54.4 | 0.98 (0.84-1.14) | 0.859 | 1.21 (1.03-1.42) | 0.018* |
| Others | 1536 | 53.4 | 1.06 (0.96-1.16) | 0.205 | 1.07 (0.97-1.19) | 0.152 | 2004 | 56.7 | 1.08 (0.99-1.17) | 0.069 | 1.14 (1.04-1.25) | 0.004* |
| Smoking status | | | | | | | | | | | | |
| Non-smoker | 4166 | 54.5 | Ref | | Ref | | 11947 | 57.9 | Ref | | Ref | |
| Current smoker | 1808 | 48.6 | 0.79 (0.73-0.85) | <0.001* | 0.81 (0.74-0.89) | <0.001* | 2858 | 52.1 | 0.79 (0.74-0.83) | <0.001* | 0.81 (0.75-0.86) | <0.001* |
| Ex-smoker | 1829 | 51.7 | 0.89 (0.82-0.96) | 0.006* | 0.92 (0.84-1.00) | 0.067 | 7916 | 53.3 | 0.83 (0.79-0.86) | <0.001* | 0.90 (0.86-0.95) | <0.001* |
| Alcohol | | | | | | | | | | | | |
| Non-drinker | 4241 | 52.2 | Ref | | Ref | | 13263 | 55.7 | Ref | | Ref | |
| Current drinker | 355 | 54.1 | 1.08 (0.92-1.26) | 0.333 | 1.10 (0.92-1.30) | 0.273 | 1123 | 58.3 | 1.11 (1.01-1.22) | 0.030* | 1.04 (0.94-1.15) | 0.365 |
| Social drinker | 2157 | 52.0 | 0.99 (0.92-1.07) | 0.905 | 1.04 (0.96-1.13) | 0.457 | 4536 | 54.3 | 0.94 (0.89-0.99) | 0.027* | 1.00 (0.95-1.06) | 0.776 |
| Ex-drinker | 522 | 50.6 | 0.94 (0.82-1.07) | 0.357 | 1.05 (0.91-1.21) | 0.289 | 1839 | 49.9 | 0.79 (0.73-0.84) | <0.001* | 1.00 (0.92-1.08) | 0.995 |
| Physical activity of moderate intensity | | | | | | | | | | | | |
| None | 4440 | 50.7 | Ref | | Ref | | 12877 | 54.5 | Ref | | Ref | |
| < 150 mins per week | 1099 | 55.3 | 1.20 (1.09-1.32) | <0.001* | 0.98 (0.89-1.09) | 0.826 | 2200 | 60.1 | 1.25 (1.17-1.34) | <0.001* | 0.96 (0.896-1.04) | 0.387 |
| At least 150 mins per week | 1683 | 58.7 | 1.38 (1.27-1.50) | <0.001* | 1.20 (1.09-1.32) | <0.001* | 6070 | 59.3 | 1.21 (1.16-1.27) | <0.001* | 1.04 (0.993-1.10) | 0.089 |
| | | | | | | | | | | | | |

| | | | | | | | | | | | | |
|---|---|---|---|---|---|---|---|---|---|---|---|---|
| Complications | | | | | | | | | | | | |
| Central obesity | | | | | | | | | | | | |
| No | 2666 | 58.3 | Ref | | Ref | | 8856 | 60.6 | Ref | | Ref | |
| Yes | 4973 | 50.0 | 0.71 (0.66-0.76) | <0.001* | 0.69 (0.64-0.75) | <0.001* | 13322 | 52.7 | 0.72 (0.69-0.75) | <0.001* | 0.72 (0.69-0.75) | <0.001* |
| Hypertension | | | | | | | | | | | | |
| No | 2661 | 52.5 | Ref | | Ref | | 4566 | 55.3 | Ref | | Ref | |
| Yes | 5151 | 52.3 | 0.99 (0.92-1.06) | 0.829 | 1.11 (0.99-1.26) | 0.071 | 18180 | 55.5 | 1.01 (0.96-1.06) | 0.700 | 1.10 (1.02-1.19) | 0.009* |
| Dyslipidaemia | | | | | | | | | | | | |
| No | 687 | 61.3 | Ref | | Ref | | 1949 | 60.7 | Ref | | Ref | |
| Yes | 6866 | 51.2 | 0.66 (0.58-0.75) | <0.001* | 0.57 (0.49-0.66) | <0.001* | 19619 | 54.5 | 0.77 (0.71-0.83) | <0.001* | 0.66 (0.60-0.73) | <0.001* |
| Stroke | | | | | | | | | | | | |
| No | 7427 | 52.3 | Ref | | Ref | | 20543 | 55.7 | Ref | | Ref | |
| Yes | 341 | 52.9 | 1.02 (0.87-1.19) | 0.780 | 0.99 (0.82-1.20) | 0.982 | 2099 | 52.5 | 0.87 (0.82-0.93) | <0.001* | 0.86 (0.52-1.40) | 0.545 |
| Coronary Heart Disease | | | | | | | | | | | | |
| No | 6932 | 52.5 | Ref | | Ref | | 18704 | 56.3 | Ref | | Ref | |
| Yes | 828 | 50.5 | 0.92 (0.83-1.02) | 0.125 | 0.93 (0.79-1.08) | 0.370 | 3925 | 51.5 | 0.82 (0.78-0.86) | <0.001* | 0.93 (0.86-1.00) | 0.057 |
| Peripheral Arterial Disease | | | | | | | | | | | | |
| No | 7118 | 52.1 | Ref | | Ref | | 20195 | 55.2 | Ref | | Ref | |
| Yes | 26 | 47.3 | 0.82 (0.48-1.40) | 0.478 | 1.03 (0.57-1.84) | 0.915 | 161 | 42.1 | 0.59 (0.48-0.72) | <0.001* | 0.92 (0.73-1.15) | 0.476 |
| Chronic Kidney disease | | | | | | | | | | | | |
| No | 3950 | 51.7 | Ref | | Ref | | 4492 | 57.1 | Ref | | Ref | |
| Yes | 3043 | 53.2 | 1.06 (0.99-1.13) | 0.080 | 1.09 (1.01-1.18) | 0.014* | 17204 | 54.9 | 0.91 (0.87-0.96) | <0.001* | 0.98 (0.93-1.04) | 0.589 |
| Malignancy | | | | | | | | | | | | |
| No | 6789 | 52.0 | Ref | | Ref | | 18247 | 55.1 | Ref | | Ref | |
| Yes | 825 | 54.5 | 1.15 (0.93-1.43) | 0.177 | 1.13 (0.90-1.42) | 0.276 | 1718 | 53.5 | 0.93 (0.87-1.00) | 0.073 | 0.97 (0.89-1.04) | 0.445 |
| Diabetic Retinopathy | | | | | | | | | | | | |
| No | 6210 | 53.8 | Ref | | Ref | | 15628 | 57.4 | Ref | | Ref | |
| Yes | 244 | 39.7 | 0.56 (0.48-0.66) | <0.001* | 0.71 (0.59-0.84) | <0.001* | 666 | 39.6 | 0.48 (0.44-0.53) | <0.001* | 0.68 (0.61-0.76) | <0.001* |
| Medications | | | | | | | | | | | | |
| Insulin treatment | | | | | | | | | | | | |
| No | 6854 | 55.7 | Ref | | Ref | | 19641 | 59.7 | Ref | | Ref | |
| Yes | 438 | 25.0 | 0.26 (0.23-0.29) | <0.001* | 0.32 (0.28-0.37) | <0.001* | 1110 | 22.5 | 0.19 (0.18-0.21) | <0.001* | 0.26 (0.24-0.28) | <0.001* |
| Antidiabetic drug | | | | | | | | | | | | |
| No | 1084 | 83.4 | Ref | | Ref | | 3755 | 84.0 | Ref | | Ref | |
| Yes | 6712 | 49.3 | 0.19 (0.16-0.22) | <0.001* | 0.22 (0.19-0.26) | <0.001* | 18933 | 51.9 | 0.20 (0.18-0.22) | <0.001* | 0.26 (0.24-0.28) | <0.001* |

| | | | | | | | | | | | |
|---|---|---|---|---|---|---|---|---|---|---|---|
| Antihypertensive drug | | | | | | | | | | | |
| No | 2297 | 52.7 | Ref | | Ref | | 3662 | 55.2 | Ref | | Ref | |
| Yes | 5016 | 51.5 | 0.95 (0.88-1.02) | 0.187 | 1.02 (0.90-1.16) | 0.734 | 17200 | 54.8 | 0.98 (0.93-1.04) | 0.597 | 1.12 (1.03-1.22) | 0.005* |
| Lipid-lowering drug | | | | | | | | | | | |
| No | 2124 | 53.7 | Ref | | Ref | | 4244 | 57.0 | Ref | | Ref | |
| Yes | 5193 | 51.2 | 0.90 (0.83-0.97) | 0.007* | 1.24 (1.13-1.36) | <0.001* | 16627 | 54.4 | 0.90 (0.85-0.94) | <0.001* | 1.22 (1.14-1.31) | <0.001* |
| Antiplatelet drug | | | | | | | | | | | |
| No | 6442 | 52.7 | Ref | | Ref | | 15983 | 57.2 | Ref | | Ref | |
| Yes | 1330 | 50.6 | 0.92 (0.85-1.00) | 0.052 | 1.08 (0.94-1.24) | 0.255 | 6666 | 51.6 | 0.80 (0.76-0.83) | <0.001* | 0.99 (0.92-1.05) | 0.796 |
| Dialysis | | | | | | | | | | | |
| No | 7720 | 52.3 | Ref | | Ref | | 22577 | 55.4 | Ref | | Ref | |
| Yes | 43 | 60.6 | 1.40 (0.87-2.26) | 0.164 | 2.44 (1.44-4.12) | 0.001* | 69 | 46.9 | 0.71 (0.51-0.98) | 0.039* | 1.70 (1.18-2.43) | 0.004* |
| Duration of Diabetes | | | | | | | | | | | |
| < 10 years | 6224 | 57.4 | Ref | | Ref | | 13218 | 64.7 | Ref | | Ref | |
| 10 years or above | 15777 | 38.8 | 0.47 (0.43-0.50) | <0.001* | 0.61 (0.56-0.66) | <0.001* | 9449 | 46.2 | 0.46 (0.45-0.48) | <0.001* | 0.65 (0.62-0.68) | <0.001* |

**Supplementary Table 3.** Factors associated with optimal control of HbA1c in females

| | Aged below 60 | | | | | | Aged 60 or above | | | | | |
|---|---|---|---|---|---|---|---|---|---|---|---|---|
| | n | Prevalence (%) | Crude odd ratio (cOR) (95% CI) | P value | Adjusted odd ratio (aOR) (95% CI) | P value | n | Prevalence (%) | Crude odd ratio (cOR) (95% CI) | P value | Adjusted odd ratio (aOR) (95% CI) | P value |
| Group | | | | | | | | | | | | |
| eHRSS only | 3923 | 52.7 | Ref | | Ref | | 18484 | 56.0 | Ref | | Ref | |
| eHealth app | 2524 | 56.5 | 1.16 (1.08-1.25) | <0.001* | 1.16 (1.07-1.26) | <0.001* | 5081 | 60.2 | 1.18 (1.13-1.24) | <0.001* | 1.13 (1.07-1.19) | <0.001* |
| Module adopted | 184 | 66.4 | 1.77 (1.37-2.29) | <0.001* | 1.65 (1.26-2.17) | <0.001* | 181 | 70.2 | 1.84 (1.41-2.40) | <0.001* | 1.49 (1.13-1.98) | 0.005* |
| Education | | | | | | | | | | | | |
| No formal education | 121 | 51.9 | Ref | | Ref | | 3460 | 55.8 | Ref | | Ref | |
| Primary | 1029 | 55.1 | 1.13 (0.86-1.49) | 0.362 | 1.15 (0.86-1.53) | 0.336 | 10429 | 56.6 | 1.03 (0.97-1.09) | 0.260 | 1.00 (0.94-1.06) | 0.993 |
| Secondary | 4487 | 53.8 | 1.07 (0.83-1.39) | 0.571 | 1.10 (0.84-1.46) | 0.465 | 8671 | 57.4 | 1.06 (1.00-1.13) | 0.032* | 1.00 (0.94-1.07) | 0.847 |
| Tertiary | 948 | 56.3 | 1.19 (0.90-1.56) | 0.213 | 1.22 (0.91-1.65) | 0.172 | 1079 | 61.0 | 1.23 (1.11-1.37) | <0.001* | 1.18 (1.05-1.32) | 0.005* |
| Occupation | | | | | | | | | | | | |
| Manual | 1265 | 54.0 | Ref | | Ref | | 1607 | 54.6 | Ref | | Ref | |
| Non-manual | 1715 | 53.3 | 0.97 (0.87-1.08) | 0.624 | 1.01 (0.90-1.14) | 0.752 | 1212 | 56.1 | 1.06 (0.95-1.19) | 0.265 | 1.10 (0.97-1.24) | 0.117 |
| Housemaker | 1854 | 56.4 | 1.10 (0.98-1.22) | 0.079 | 1.20 (1.07-1.34) | 0.001* | 12947 | 58.4 | 1.16 (1.08-1.26) | <0.001* | 1.28 (1.181.392 | <0.001* |
| Retired | 147 | 53.6 | 0.98 (0.76-1.26) | 0.915 | 1.06 (0.81-1.39) | 0.630 | 5736 | 57.7 | 1.13 (1.04-1.23) | 0.003* | 1.22 (1.11-1.33) | <0.001* |
| Unemployed | 280 | 50.8 | 0.88 (0.73-1.06) | 0.179 | 1.03 (0.85-1.26) | 0.720 | 159 | 55.0 | 1.01 (0.79-1.29) | 0.883 | 1.13 (0.87-1.46) | 0.353 |
| Others | 949 | 57.7 | 1.16 (1.02-1.31) | 0.022* | 1.15 (1.01-1.32) | 0.034* | 855 | 59.7 | 1.23 (1.08-1.40) | 0.001* | 1.25 (1.09-1.43) | 0.001* |
| Smoking status | | | | | | | | | | | | |
| Non-smoker | 5944 | 55.3 | Ref | | Ref | | 22853 | 57.2 | Ref | | Ref | |
| Current smoker | 363 | 47.3 | 0.72 (0.62-0.84) | <0.001* | 0.77 (0.66-0.91) | <0.001* | 252 | 50.3 | 0.75 (0.63-0.90) | 0.002* | 0.80 (0.66-0.96) | 0.022* |
| Ex-smoker | 313 | 47.3 | 0.72 (0.61-0.84) | <0.001* | 0.85 (0.71-1.01) | 0.072 | 605 | 53.2 | 0.85 (0.75-0.95) | 0.007* | 1.04 (0.84-1.00) | 0.482 |
| Alcohol | | | | | | | | | | | | |
| Non-drinker | 5348 | 54.9 | Ref | | Ref | | 20146 | 57.0 | Ref | | Ref | |
| Current drinker | 44 | 62.0 | 1.33 (0.82-2.16) | 0.236 | 1.39 (0.83-2.33) | 0.201 | 76 | 55.9 | 0.95 (0.68-1.34) | 0.791 | 0.83 (0.58-1.26) | 0.340 |
| Social drinker | 667 | 49.9 | 0.81 (0.72-0.91) | <0.001* | 0.86 (0.76-0.98) | 0.025* | 1106 | 53.1 | 0.85 (0.78-0.93) | 0.001* | 0.87 (0.79-0.96) | 0.008* |
| Ex-drinker | 121 | 46.4 | 0.70 (0.55-0.90) | 0.006* | 0.90 (0.68-1.18) | 0.460 | 225 | 44.9 | 0.61 (0.51-0.73) | <0.001* | 0.80 (0.65-0.97) | 0.027* |
| Physical activity of moderate intensity | | | | | | | | | | | | |
| None | 4232 | 53.4 | Ref | | Ref | | 15316 | 56.7 | Ref | | Ref | |
| < 150 mins per week | 848 | 58.3 | 1.22 (1.08-1.33) | 0.001* | 0.98 (0.87-1.11) | 0.806 | 2130 | 62.4 | 1.26 (1.17-1.36) | <0.001* | 0.94 (0.86-1.01) | 0.124 |
| At least 150 mins per week | 1151 | 59.3 | 1.27 (1.15-1.40) | <0.001* | 1.07 (0.96-1.19) | 0.209 | 5087 | 60.3 | 1.16 (1.10-1.22) | <0.001* | 1.03 (0.97-1.08) | 0.284 |

| | | | | | | | | | | | |
|---|---|---|---|---|---|---|---|---|---|---|---|
| Complications | | | | | | | | | | | |
| Central obesity | | | | | | | | | | | |
| No | 1293 | 61.8 | Ref | | Ref | | 4428 | 63.9 | Ref | | Ref | |
| Yes | 5224 | 53.1 | 0.69 (0.63-0.77) | <0.001* | 0.71 (0.64-0.79) | <0.001* | 18674 | 55.7 | 0.70 (0.67-0.74) | <0.001* | 0.71 (0.67-0.75) | <0.001* |
| Hypertension | | | | | | | | | | | |
| No | 2754 | 55.0 | Ref | | Ref | | 4632 | 57.0 | Ref | | Ref | |
| Yes | 3877 | 54.0 | 0.95 (0.89-1.03) | 0.255 | 1.03 (0.90-1.18) | 0.587 | 19114 | 57.0 | 1.00 (0.95-1.05) | 0.969 | 1.21 (1.11-1.31) | <0.001* |
| Dyslipidaemia | | | | | | | | | | | |
| No | 541 | 60.5 | Ref | | Ref | | 1353 | 62.4 | Ref | | Ref | |
| Yes | 5916 | 53.7 | 0.75 (0.65-0.87) | <0.001* | 0.61 (0.51-0.71) | <0.001* | 21330 | 56.3 | 0.77 (0.71-0.84) | <0.001* | 0.64 (0.57-0.71) | <0.001* |
| Stroke | | | | | | | | | | | |
| No | 6394 | 54.4 | Ref | | Ref | | 21799 | 57.2 | Ref | | Ref | |
| Yes | 193 | 53.6 | 0.97 (0.78-1.19) | 0.781 | 1.11 (0.86-1.44) | 0.405 | 1809 | 54.2 | 0.88(0.82-0.95) | 0.001* | 1.05 (0.96-1.15) | 0.262 |
| Coronary Heart Disease | | | | | | | | | | | |
| No | 6368 | 54.3 | Ref | | Ref | | 21340 | 57.5 | Ref | | Ref | |
| Yes | 216 | 56.3 | 1.08 (0.88-1.32) | 0.444 | 1.46 (1.12-1.91) | 0.005* | 2252 | 52.3 | 0.81 (0.76-0.86) | <0.001* | 1.05 (0.96-1.14) | 0.261 |
| Peripheral Arterial Disease | | | | | | | | | | | |
| No | 6054 | 54.3 | Ref | | Ref | | 20992 | 56.7 | Ref | | Ref | |
| Yes | 12 | 40.0 | 0.56 (0.27-1.16) | 0.121 | 0.56 (0.26-1.21) | 0.143 | 136 | 46.4 | 0.66 (0.52-0.83) | <0.001* | 1.17 (0.91-1.51) | 0.214 |
| Chronic Kidney disease | | | | | | | | | | | |
| No | 4132 | 53.7 | Ref | | Ref | | 5840 | 57.9 | Ref | | Ref | |
| Yes | 1621 | 55.4 | 1.07 (0.98-1.17) | 0.104 | 1.21 (1.10-1.33) | <0.001* | 16528 | 56.7 | 0.95 (0.91-0.99) | 0.042* | 1.12 (1.06-1.18) | <0.001* |
| Malignancy | | | | | | | | | | | |
| No | 5578 | 54.2 | Ref | | Ref | | 18601 | 56.7 | Ref | | Ref | |
| Yes | 416 | 54.5 | 1.01 (0.87-1.17 | 0.869 | 0.98 (0.84-1.15) | 0.888 | 2379 | 56.7 | 1.00 (0.94-1.07) | 0.936 | 1.04 (0.97-1.12) | 0.207 |
| Diabetic Retinopathy | | | | | | | | | | | |
| No | 5223 | 55.5 | Ref | | Ref | | 15867 | 59.2 | Ref | | Ref | |
| Yes | 111 | 31.8 | 0.37 (0.29-0.47) | <0.001* | 0.51 (0.40-0.65) | <0.001* | 516 | 36.5 | 0.39 (0.35-0.44) | <0.001* | 0.64 (0.56-0.72) | <0.001* |
| Medications | | | | | | | | | | | |
| Insulin treatment | | | | | | | | | | | |
| No | 5855 | 57.8 | Ref | | Ref | | 20493 | 61.1 | Ref | | Ref | |
| Yes | 331 | 25.5 | 0.24 (0.21-0.28) | <0.001* | 0.32 (0.27-0.37) | <0.001* | 1017 | 22.6 | 0.18 (0.17-0.20) | <0.001* | 0.27 (0.25-0.29) | <0.001* |
| Antidiabetic drug | | | | | | | | | | | |
| No | 1316 | 82.8 | Ref | | Ref | | 4678 | 84.0 | Ref | | Ref | |
| Yes | 5299 | 50.1 | 0.20 (0.18-0.23) | <0.001* | 0.24 (0.20-0.27) | <0.001* | 18995 | 52.8 | 0.21 (0.19-0.22) | <0.001* | 0.28 (0.26-0.31) | <0.001* |
| Antihypertensive drug | | | | | | | | | | | |
| No | 2500 | 55.2 | Ref | | Ref | | 4185 | 58.2 | Ref | | Ref | |

| | | | | | | | | | | | | |
|---|---|---|---|---|---|---|---|---|---|---|---|---|
| Yes | 3706 | 53.5 | 0.93 (0.86-1.00) | 0.061 | 1.00 (0.87-1.15) | 0.980 | 17493 | 56.3 | 0.92 (0.87-0.97) | 0.003* | 0.96 (0.84-1.05) | 0.397 |
| Lipid-lowering drug | | | | | | | | | | | | |
| No | 2135 | 55.7 | Ref | | Ref | | 4066 | 58.1 | Ref | | Ref | |
| Yes | 4078 | 53.4 | 0.91 (0.84-0.98) | 0.018* | 1.24 (1.13-1.36) | <0.001* | 17596 | 56.3 | 0.93 (0.88-0.98) | 0.006* | 1.23 (1.15-1.31) | <0.001* |
| Antiplatelet drug | | | | | | | | | | | | |
| No | 6107 | 54.7 | Ref | | Ref | | 18924 | 58.5 | Ref | | Ref | |
| Yes | 490 | 50.7 | 0.85 (0.74-0.97) | 0.016* | 0.86 (0.71-1.05) | 0.145 | 4709 | 51.4 | 0.75 (0.71-0.78) | <0.001* | 0.85 (0.79-0.91) | <0.001* |
| Dialysis | | | | | | | | | | | | |
| No | 6569 | 54.3 | Ref | | Ref | | 23569 | 57.0 | Ref | | Ref | |
| Yes | 18 | 72.0 | 2.16 (0.90-5.18) | 0.084 | 5.20 (2.04-13.25) | 0.001* | 46 | 52.3 | 0.82 (0.54-1.25) | 0.376 | 2.63 (1.66-4.18) | 0.001* |
| Duration of Diabetes | | | | | | | | | | | | |
| < 10 years | 5288 | 59.3 | Ref | | Ref | | 13634 | 68.0 | Ref | | Ref | |
| 10 years or above | 1338 | 41.1 | 0.34 (0.11-1.00) | 0.051 | 0.69 (0.63-0.76) | <0.001* | 10035 | 46.7 | 0.41 (0.39-0.42) | <0.001* | 0.59 (0.57-0.62) | <0.001* |

**Supplementary figure 1.** Flow chart of participants selection of the study

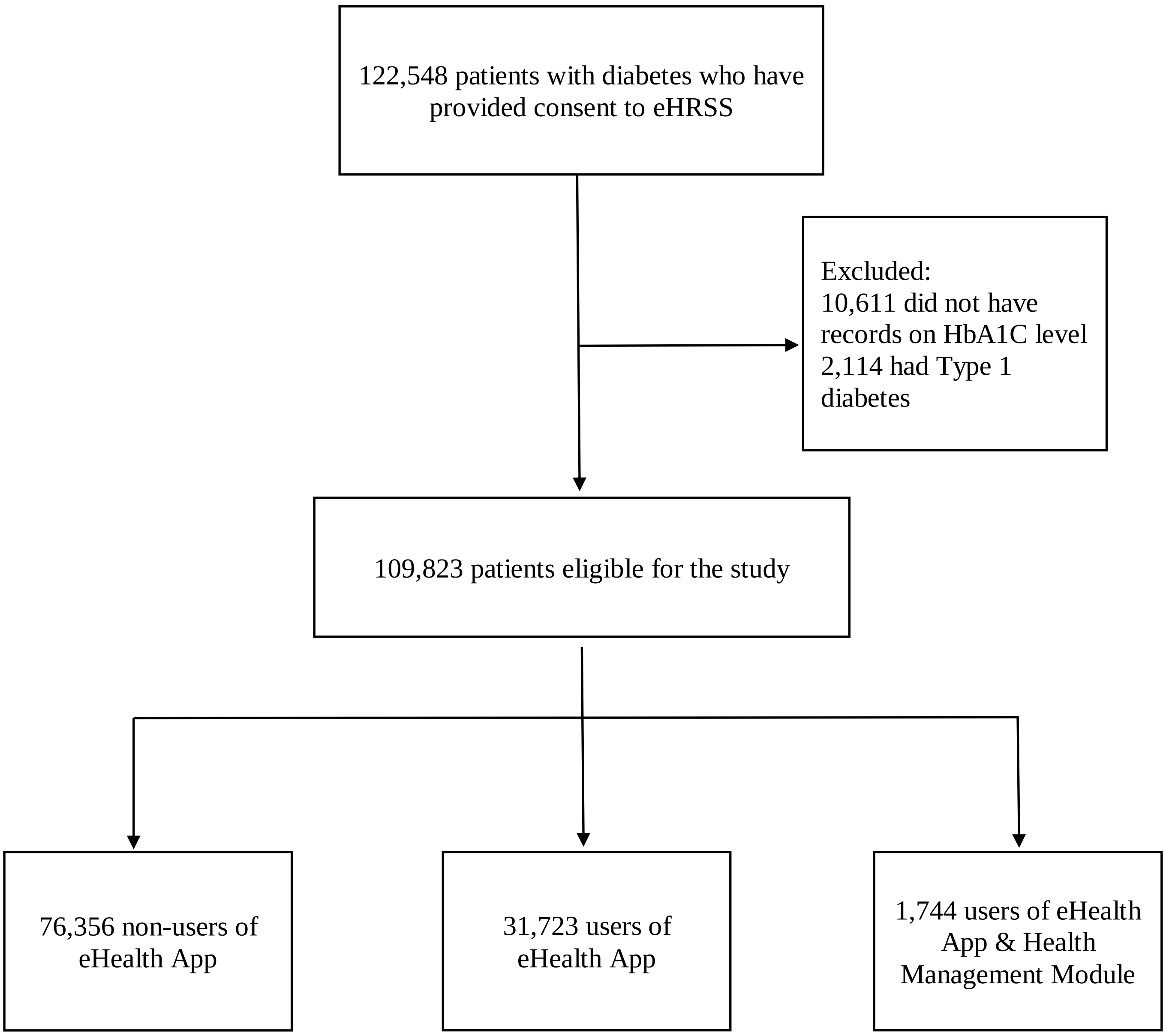

Supplement: Supplementary file 1 — Supplementary info [file 41746_2023_807_MOESM1_ESM.pdf]
